# Impact of *In Vitro* Long-Term Low-Level DEHP Exposure on Gene Expression Profile in Human Granulosa Cells

**DOI:** 10.3390/cells11152304

**Published:** 2022-07-27

**Authors:** Dragana Samardzija Nenadov, Kristina Pogrmic-Majkic, Biljana Tesic, Dunja Kokai, Svetlana Fa Nedeljkovic, Bojana Stanic, Nebojsa Andric

**Affiliations:** Department of Biology and Ecology, Faculty of Sciences, University of Novi Sad, 21000 Novi Sad, Serbia; dragana.samardzija@dbe.uns.ac.rs (D.S.N.); biljana.tesic@dbe.uns.ac.rs (B.T.); dunja.kokai@dbe.uns.ac.rs (D.K.); svetlana.fa@dbe.uns.ac.rs (S.F.N.); bojana.stanic@dbe.uns.ac.rs (B.S.); nebojsa.andric@dbe.uns.ac.rs (N.A.)

**Keywords:** granulosa cells, di(2-ethylhexyl) phthalate, steroidogenesis, RNA sequencing, SULT1A3/4

## Abstract

Here, we applied a model of long-term exposure of human granulosa cells to low environmentally relevant levels of di(2-ethylhexyl) phthalate (DEHP). This approach provides more relevant data regarding the impact of DEHP on the function of human granulosa cells. The immortalized human granulosa cells HGrC1 were exposed to 50 nM and 250 nM DEHP for four weeks. The cells were collected every week to analyze the basal granulosa cells’ functions. A portion of the DEHP-exposed cells was stimulated with forskolin (FOR) for 48 h. Steroidogenesis was investigated using ELISA, whereas DNBQ sequencing and RT-qPCR were used to analyze gene expression. The results show that steroidogenesis was not affected by DEHP exposure. RNAsequencing shows that DEHP caused week- and concentration-specific changes in various genes and functions in HGrC1. Sulfotransferase family 1A member 3 (*SULT1A3*) and 4 (*SULT1A4*), which are involved in catecholamine metabolism, were the most prominent genes affected by DEHP under both the basal and FOR-stimulated conditions in all four weeks of exposure. This study showed, for the first time, that *SULT1A3* and *SULT1A4* are expressed in human granulosa cells, are regulated by FOR, and are affected by low-level DEHP exposure. These data provide new insight into the relationship between DEHP, *SULT1A3*, and *SULT1A4* in human granulosa cells.

## 1. Introduction

Infertility is a medical condition that affects an estimated 10–12% of the reproductive-age couples worldwide, with female infertility factors contributing to approximately 35–40% of all cases [1]. In addition to the common risk factors such as age, genetics, and lifestyle, exposure to endocrine disruptors (EDs) has been highlighted as an important risk factor that contributes to female infertility. These assumptions are supported by the fact that many EDs and their metabolites have been found in women’s follicular fluid [2,3,4,5].

One of the EDs with a potential negative impact on women’s reproductive health is di(2-ethylhexyl) phthalate (DEHP). It is the most widely used plasticizer and is found in a variety of products such as construction material, clothing, and furniture. DEHP is not covalently bound to the plastic; hence, it can be easily discharged into the environment during the use of plastic products. Humans are exposed to DEHP through oral ingestion, inhalation, and through the skin [4]. DEHP and its metabolites are found in women’s follicular fluid in nM concentrations, and their presence in the follicular fluid has been associated with different reproductive disorders, such as polycystic ovary syndrome [5], poor ovarian reserve [6], and altered intrafollicular reproductive hormones in women undergoing in vitro fertilization (IVF) [7].

Granulosa cells of the ovarian follicle play an essential role in the proper functioning of the female reproductive system due to the production of reproductive hormones as well as growth factors that interact with the oocyte during the growth and development of the follicle [8]. Animal models have shown that DEHP has an adverse effect on ovarian steroidogenesis in in vivo studies on rodents [9,10] and also in in vitro studies using rat granulosa cells [11]. Although animal studies are important for a better understanding of the effect DEHP exerts on ovarian function, the data obtained from those studies cannot be easily translated to humans. The availability of several human granulosa cell lines and granulosa cells from the IVF procedure foster the investigation of the effects of DEHP in human models. Such investigations have shown that DEHP decreases estradiol production in the follicle-stimulating hormone (FSH)-stimulated human granulosa-like tumor cell line KGN [12], lowers progesterone biosynthesis [13], and changes the levels of androgens but not estradiol in primary human granulosa cells and the KGN cell line [5]. Besides steroidogenesis, DEHP lowers cell viability, promotes cell cycle arrest and apoptosis, and alters the expression of apoptosis-related genes in the primary culture of human granulosa cells [5]. However, these studies evaluated a short-term exposure to DEHP (up to 72 h), which does not precisely represent a real-life human exposure scenario where granulosa cells are continuously exposed to low levels of DEHP present in human follicular fluid. The cellular responses following a long-term exposure to DEHP may be distinct from the responses to a short-term exposure. Moreover, short-term exposure studies cannot capture a possible adaptive response in target cells that might occur during a long-term exposure. To gain insight into the long-term effects of EDs in human cells, a limited number of studies employed immortalized cell lines that may be exposed to EDs for longer periods of time. Some of these studies revealed a distinct effect on steroidogenesis following a long-term exposure of human granulosa cells to the mixture of EDs [14], an ED-specific transcriptional reprograming in human breast cancer cells [15], dynamic, time-dependent changes in the response of human bronchial epithelial cells to the total particulate matter from a candidate modified-risk tobacco product [16], or a specific bisphenol-A-mediated effect in human vascular endothelial cells [17]. Moreover, long-term exposure studies also help in better understanding the biological effects of the relevant levels of several EDs in trophoblasts [18] or the molecular events involved in malignant transformation [19].

In the present study, our objective was to determine whether a long-term exposure to low, “real-life” concentrations of DEHP has an adverse effect on the function of human granulosa cells. For this purpose, we employed a four-week-long exposure of HGrC1 human granulosa cells to 50 nM and 250 nM DEHP (referred to as DEHP50 and DEHP250). It has been shown that DEHP can exert a negative effect on human granulosa cells in nanomolar concentrations [5,13]. This is the reason why we decided to use nanomolar concentrations of DEHP in this study. Although the concentration of DEHP in human follicular fluid was shown to be 1.21 ng/mL (~3 nM) [5], we had started the long-term experiments before this result was reported. Therefore, the concentrations of DEHP were selected based on the literature data available at that moment. DEHP50 was chosen based on the concentration of its metabolite mono(2-ethylhexyl) phthalate detected in human follicular fluid [20], whereas DEHP250 was selected based on the concentration detected in human serum [21]. The human granulosa cell line HGrC1 was chosen for the study since these cells are immortalized and display the characteristics of human granulosa cells belonging to the early-stage follicles [22]. To our knowledge, this is the first study that provides an in-depth analysis of the effect of long-term low-level DEHP exposure in human granulosa cells, thereby providing a more relevant picture of the impact of DEHP on human reproductive health.

## 2. Materials and Methods

### 2.1. Chemicals

Dulbecco’s Modified Eagle’s Medium/Nutrient Mixture F-12 Ham (DMEM/F12), dimethyl sulfoxide (DMSO), DEHP, penicillin (10,000 IU/mL)–streptomycin (10 mg/mL) mixture, 0.25% trypsin-EDTA solution, and Roche Complete EDTA-free protease inhibitor cocktail tablets were obtained from Sigma-Aldrich Company (Steinheim, Germany). Forskolin (FOR) was obtained from Abcam (Cambridge, UK). Fetal bovine serum (FBS) was obtained from Capricorn Scientific GmbH (Ebsdorfergrund, Germany). A High-Capacity cDNA Reverse Transcription Kit and Power SYBR Green PCR Master Mix were from Applied Biosystems (Foster City, CA, USA). TRIzol Reagent, alamarBlue Cell Viability Reagent, SuperSignal West Femto Maximum Sensitivity Substrate, and Pierce BCA Protein Assay Kit were purchased from Thermo Fisher Scientific (Waltham, MA, USA). CSL-BBL Pre-stained Protein Standard was obtained from Cleaver Scientific (Warwickshire, UK). Estradiol and Progesterone ELISA Kits were obtained from Cayman Chemicals (Ann Arbor, MI, USA). Anti-aromatase primary antibody was purchased from Abcam (Cambridge, UK), whereas the primary antibodies against steroidogenic acute regulatory protein (STAR) and glyceraldehyde 3-phosphate dehydrogenase (GAPDH) were from Cell Signaling Technology (Danvers, MA, USA). Horseradish peroxidase (HRP)-linked secondary anti-rabbit antibody was from Bio-Rad (Hercules, CA, USA). All other chemicals were of analytical grade.

### 2.2. Culture of HGrC1 Cells and the Long-Term Exposure Study

**Figure 1:**
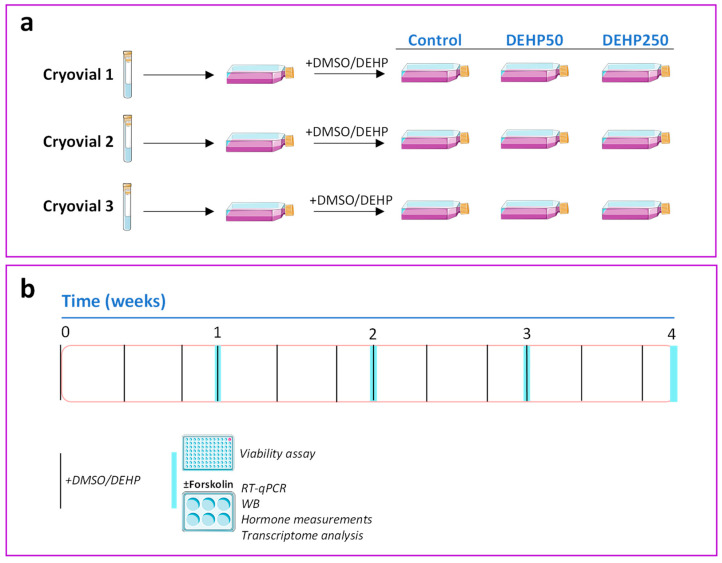
Experimental design of the long-term exposure of HGrC1 cells to DEHP. (**a**) Three different cryopreserved stock vials of HGrC1 cells were thawed into three 25 cm^2^ cell culture flasks and cultured for two weeks, after which the cells from each flask were divided into three 75 cm^2^ flasks. During the next four weeks, HGrC1 cells were subcultured twice a week and exposed to either vehicle (0.05% DMSO-control) or DEHP (50 nM or 250 nM in 0.05% DMSO) three times a week. (**b**) Different endpoint measurements were taken after 1, 2, 3, and 4 weeks of exposure. Pictures were obtained from Servier Medical Art (https://smart.servier.com/).

Human HGrC1 non-luteinized granulosa cells were kindly provided by Dr. A. Iwase (Nagoya University, Japan). The HGrC1 cells were cultured as described in [14]. For the long-term exposure study, three different cryopreserved stock vials of HGrC1 cells belonging to different passages (biological replicates) were thawed into three 25 cm^2^ cell culture flasks and cultured for two weeks, after which the cells from each flask were divided into three 75 cm^2^ flasks. Three hours after plating, either vehicle (0.05% DMSO) or DEHP (50 nM or 250 nM in 0.05% DMSO) were added to the flasks. After that, the cells were subcultured twice a week, on Tuesdays and Fridays, with 2.25 × 10^6^ and 1.35 × 10^6^ cells returned to each flask, respectively. The treatments were added to the culture flasks three hours after plating the cells back into the flasks to avoid the effect of DEHP on cell attachment to the flask surface. In addition, treatments were added to the appropriate cell culture flasks on Sundays as well, thus enabling a repeated, long-term exposure to DEHP. Cells were treated as described above for four weeks. After 1, 2, 3, and 4 weeks of the repeated exposure, the cells were plated into cell culture plates for different endpoint measurements. For the cell viability assay, the cells derived from the control and DEHP flasks were plated into 96-well plates (1 × 10^5^ cells/well) after 1, 2, 3, and 4 weeks of the repeated DEHP exposure. Three hours after plating, the cells were exposed to either vehicle or two concentrations of DEHP for an additional 48 h. For hormone assays, quantitative reverse transcription PCR (RT-qPCR), Western blotting, and transcriptome analysis, the cells derived from the control and DEHP flasks were plated into wells of a 6-well plate (0.75 × 10^6^ cells/well) after 1, 2, 3, and 4 weeks of the repeated DEHP exposure. Three hours after plating, the cells were exposed to either vehicle or two concentrations of DEHP. Then, 24h after plating, some cells were stimulated with forskolin (25 µM) for an additional 48 h (referred to as FOR-stimulated HGrC1 cells). For the estradiol measurements, androstenedione (10 µM) was added to the cell culture medium as a substrate for aromatase. A schematic representation of the experimental design of the long-term exposure study is provided in Figure 1.

### 2.3. Culture of Human Cumulus Granulosa Cells

Human cumulus granulosa cells were obtained from women undergoing IVF procedures at the Clinic for Gynecology and Obstetrics, Clinical Center of Vojvodina, Novi Sad, Serbia. The study was approved by the Ethics Committee of the Clinical Center of Vojvodina (approval number: 00-313), and signed informed consent was obtained from each participant. The exclusion criteria and the protocol for obtaining and isolating granulosa cells are described in [23].

### 2.4. Morphological Analysis

The morphology of the cells was observed in culture flasks after 1, 2, 3, and 4 weeks of the repeated exposure using an Olympus IX51 inverted microscope (100× magnification), and photographs were taken.

### 2.5. Cell Viability Assay

An Alamar Blue assay was performed to quantify the viability of HGrC1 cells following exposure to DEHP. The assay was performed according to the manufacturer’s instructions: First, 10% Alamar Blue was added to the cell culture medium and the plates were incubated in the dark for 2 h at 37 °C. The resulting fluorescence was measured on a Thermo Labsystems Fluoroskan Ascent fluorescence plate reader with the following settings: excitation wavelength 540 nm, emission wavelength 590 nm.

### 2.6. Hormone Measurements

Cell culture media were collected and stored at −20 °C. The estradiol and progesterone levels accumulated in the incubation media were analyzed using the Estradiol and Progesterone ELISA Kits, according to the manufacturer’s instructions. The results are expressed as pg/mg of protein. The protein concentrations in the cell lysates were determined using the Pierce BCA Protein Assay Kit.

### 2.7. mRNA Sequencing

For the mRNA sequencing (RNAseq) analysis, the cells were collected in TRIzol and stored at −80 °C until RNA isolation. Extracted RNAs from three independent experiments were pooled and submitted to BGI (BGI Europe, Copenhagen, Denmark) for the RNAseq analysis. The quantity and integrity of RNA was assessed using an Agilent 4200 Bioanalyzer (Agilent Technologies, Santa Clara, CA, USA). All samples had an RNA integrity number > 9.1 and were of very high quality. The RNAseq was performed on the DNBSEQ platform. The sample reads were trimmed to remove reads with an unknown base (N) content greater than 5% and adapters and low-quality bases aligned with the reference genome and genes using HISAT and Bowtie2 software, respectively. A bioinformatic analysis was performed by BGI (BGI Europe, Copenhagen, Denmark).

### 2.8. Quantitative Reverse Transcription PCR (RT-qPCR) Analysis

The mRNA expression analysis was performed using RT-qPCR. The cells were collected in TRIzol and stored at −80 °C until RNA isolation. The extracted RNA was transcribed into cDNA using a High-Capacity cDNA Reverse Transcription Kit, and qPCR was performed using Power SYBR Green PCR Master Mix on the Mastercycler RealPlex (Eppendorf, Hamburg, Germany) real-time PCR system. The collected data were processed with a comparative cycle threshold (∆∆Ct) method with an automatically adjusted fluorescence threshold (ΔRn). A complete list of primers and their sequences is given in Table 1. The treatments had no effect on *GAPDH* expression.

### 2.9. Western Blot Analysis

Western blot analysis was used to investigate protein expression after the long-term exposure of HGrC1 cells to DEHP. The analysis was performed as described in [14]. The dilution for the aromatase and STAR primary antibodies was 1:1000, and the dilution was 1:3000 for the GAPDH primary antibody. The HRP-linked secondary antibody was diluted 1:3000. The signals were visualized using a myECL imager (Thermo Scientific, Chicago, IL, USA) and were quantified using the NIH ImageJ software [24].

### 2.10. Statistical Analysis

Statistical comparisons were performed by a one-way analysis of variance (ANOVA) with Dunnet’s or Tukey’s multiple comparison posthoc test, where appropriate, using the Prism 8 software package (GraphPad Software, Inc., La Jolla, CA, USA). A *p* value of <0.05 was considered significant.

## 3. Results

### 3.1. Effects of the Long-Term Low-Level DEHP Exposure on Viability and Steroidogenesis of HGrC1 Cells

**Figure 2:**
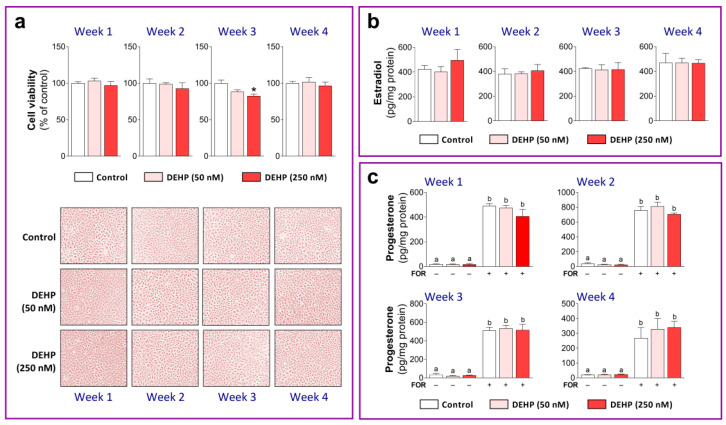
Viability and steroidogenesis in DEHP-exposed HGrC1 cells. (**a**) Cell viability was assessed using the Alamar Blue assay. Results are expressed relative to the vehicle-treated control, which was set as 100% in each week. Representative images of the control and DEHP-exposed HGrC1 cells after 1, 2, 3, and 4 weeks are shown. (**b**) Estradiol and (**c**) progesterone production in the culture medium were measured using ELISA. Each data point represents the mean ± SEM of three independent experiments. * *p* < 0.05 vs. control. Different superscript letters indicate statistically significant differences among treatment groups (*p* < 0.05).

First, we analyzed the viability of HGrC1 cells after 1, 2, 3, and 4 weeks of the repeated DEHP exposure. Only DEHP250 slightly decreased cell viability after 3 weeks of exposure, which returned to control values after 4 weeks of exposure. We did not notice any morphological changes in HGrC1 cells after repeated DEHP exposure (Figure 2a). Next, we analyzed the effect of DEHP on steroidogenesis in HGrC1 cells. It has been reported that FSH and FOR (an adenylyl cyclase activator) could not induce aromatase (CYP19A1) mRNA expression or estradiol synthesis in HGrC1 cells. Progesterone production and the expression of STAR can be induced only after FOR stimulation [14]. Therefore, the effects of DEHP50 and DEHP250 in the current study were analyzed only at the level of basal estradiol production, whereas progesterone production was analyzed under the basal and FOR-stimulated conditions. The results show that both concentrations of DEHP had no effect on estradiol production after 1, 2, 3, and 4 weeks of exposure (Figure 2b). Long-term exposure to DEHP did not affect the basal and FOR-stimulated production of progesterone in HGrC1 cells during any investigated week (Figure 2c). The levels of proteins involved in the production of estradiol and progesterone, CYP19A1 and STAR, were also not changed after the long-term low-level DEHP exposure (Supplementary Figure S1).

### 3.2. Effects of the Long-Term Low-Level DEHP Exposure on HGrC1 Transcriptome under the Basal and FOR-Stimulated Conditions

**Figure 3:**
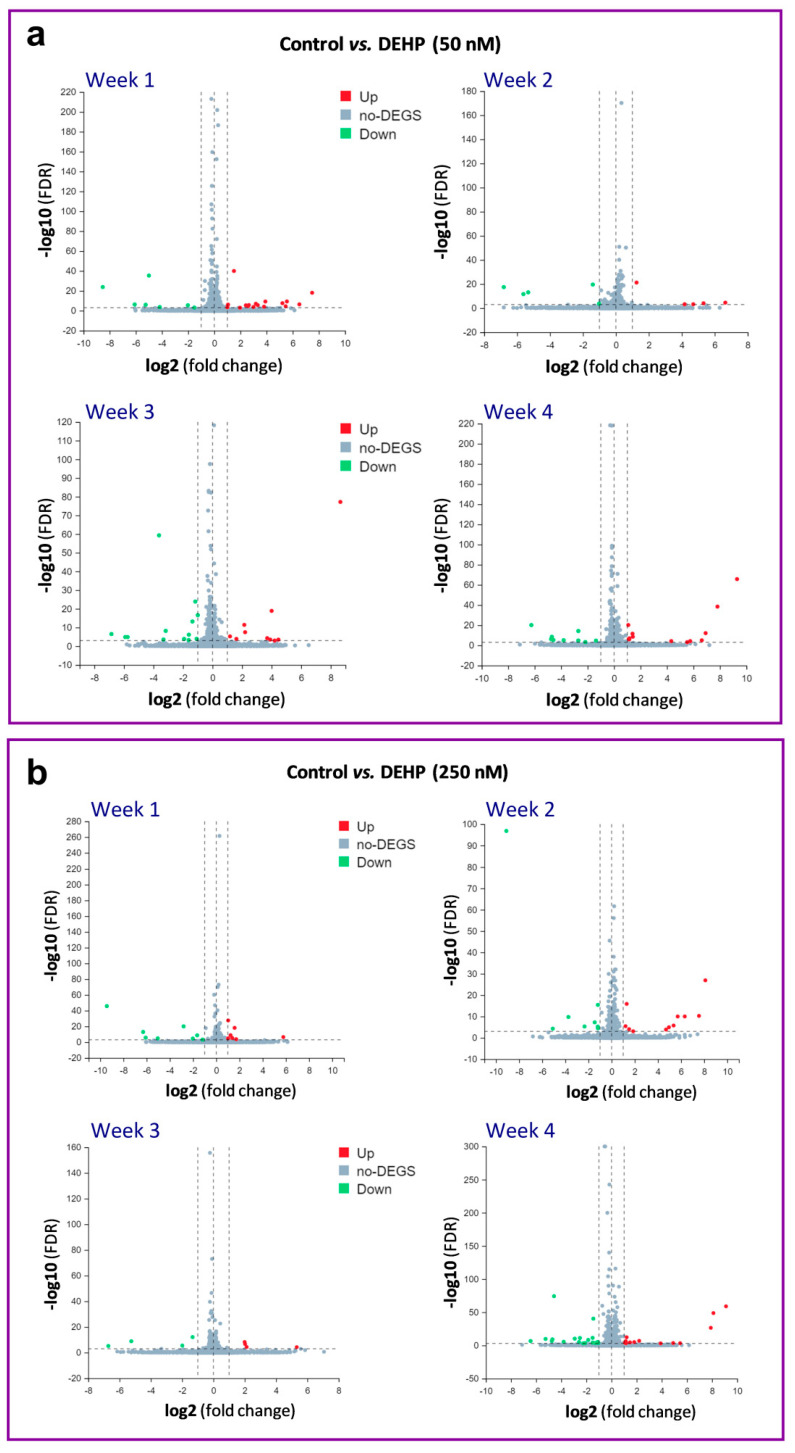
Volcano scatter plots of mRNA expression in DEHP-exposed HGrC1 cells. (**a**) DEHP50 and (**b**) DEHP250 compared to the non-treated control. The X-axis represents the fold change of the difference after conversion to log2, and the Y-axis represents the significance value (false discovery rate, FDR) after conversion to −log10. In each plot, significantly upregulated genes are highlighted in red, and downregulated genes are highlighted in green. Non-significant findings are represented as grey dots.

**Figure 4:**
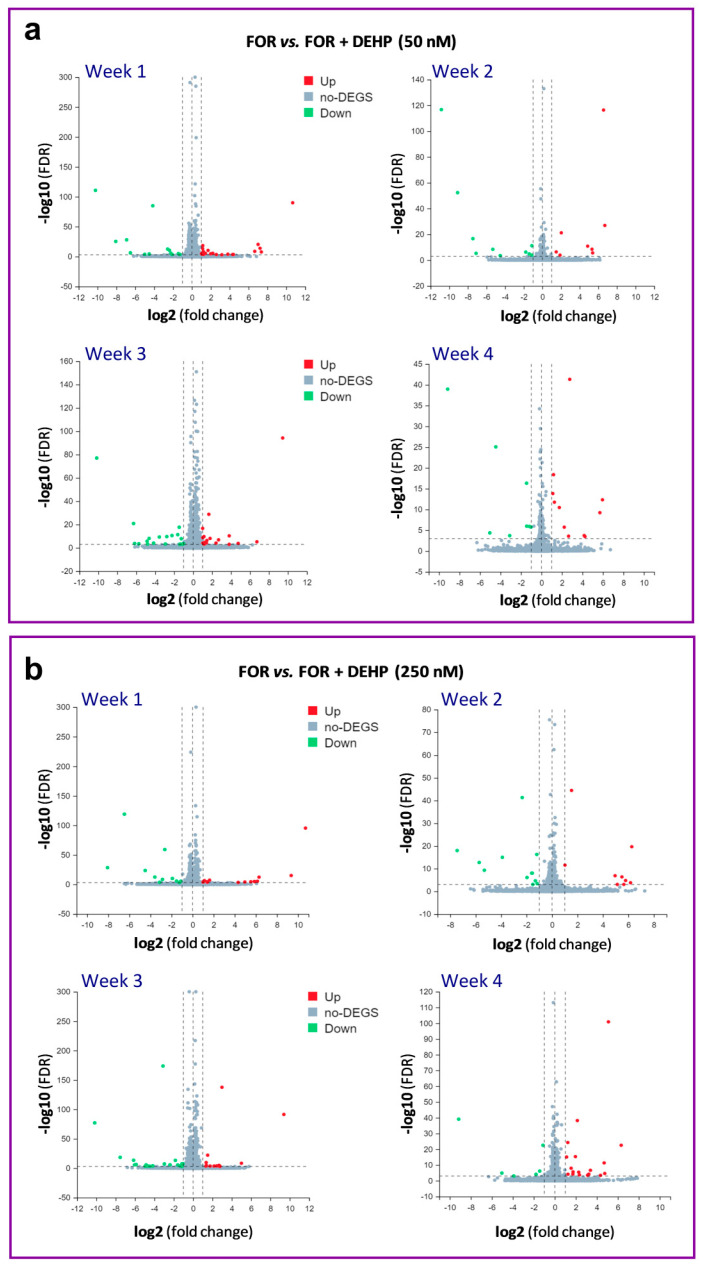
Volcano scatter plots of mRNA expression in DEHP-exposed HGrC1 cells stimulated with FOR. (**a**) DEHP50 and (**b**) DEHP250 compared to the FOR-stimulated group. The X-axis represents the fold change of the difference after conversion to log2, and the Y-axis represents the significance value (false discovery rate, FDR) after conversion to −log10. In each plot, significantly upregulated genes are highlighted in red, and downregulated genes are highlighted in green. Non-significant findings are represented as grey dots.

Since steroidogenesis in HGrC1 cells was not affected by the long-term low-level DEHP exposure, we performed a whole-genome transcriptome analysis to reveal the potential molecular targets of DEHP after the long-term exposure. The transcriptome analysis revealed differentially expressed genes (DEGs) in HGrC1 cells with at least 2-fold changes and false discovery rates (FDRs) ≤0.001 after 1, 2, 3, and 4 weeks of the repeated exposure to DEHP50 and DEHP250 under the basal (Figure 3a,b) and FOR-stimulated conditions (Figure 4a,b). The results show that the highest number of DEGs was associated with 4 weeks of exposure to DEHP250 (*n* = 33), with 14 upregulated and 19 downregulated genes. In FOR-stimulated HGrC1 cells, the highest number of DEGs was associated with 3 weeks of exposure to DEHP50 (*n* = 37), with 18 upregulated and 19 downregulated genes. Considering all 4 weeks of exposure, DEHP50 and DEHP250 together upregulated 81 genes and downregulated 73 genes under basal conditions, whereas both treatments upregulated 111 and downregulated 99 genes under FOR-stimulated conditions.

**Figure 5:**
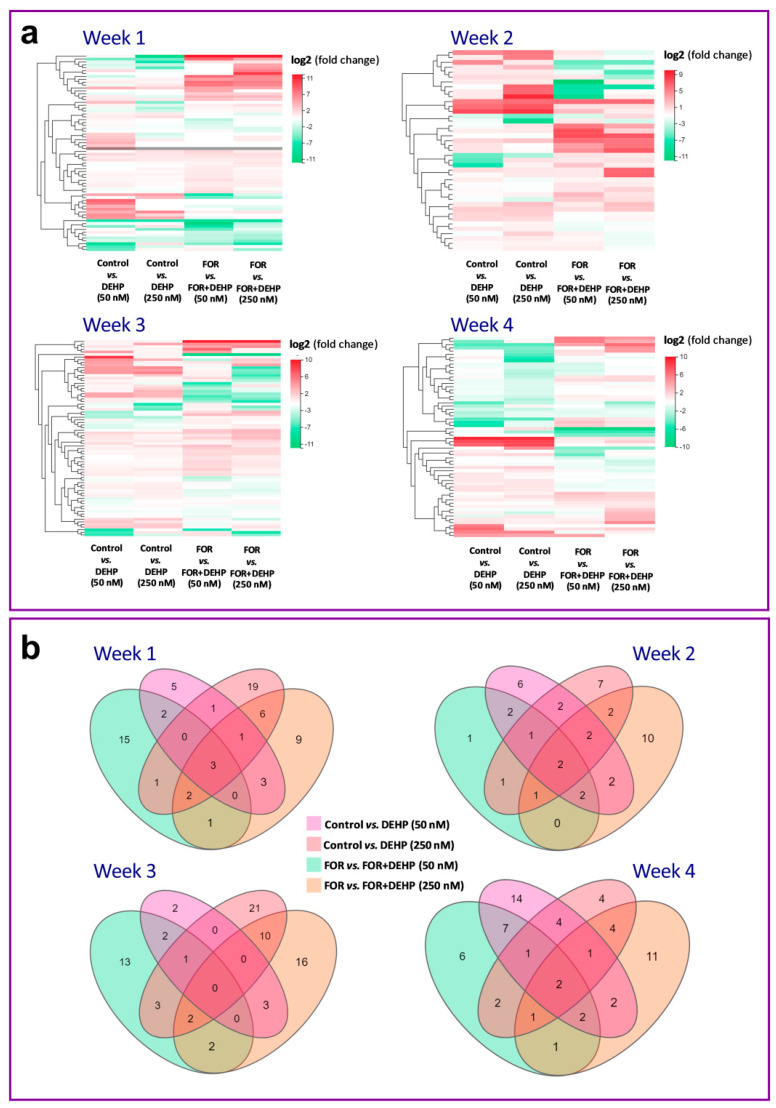
Heatmap and Venn diagrams of deregulated genes in DEHP-exposed HGrC1 cells under the basal and FOR-stimulated conditions. (**a**) Cluster analysis of significantly changed transcripts after 1, 2, 3, and 4 weeks of the repeated DEHP50 and DEHP250 exposure under the basal and FOR-stimulated conditions. Upregulated genes are highlighted in red, and downregulated genes are highlighted in green, whereas those that remained unchanged are in white. (**b**) Venn diagrams show overlapping deregulated genes after 1, 2, 3, and 4 weeks of the repeated DEHP50 and DEHP250 exposures under the basal and FOR-stimulated conditions.

The results of the hierarchical clustering of DEGs show distinct expression clusters characteristic of the different treatment conditions (basal and FOR), DEHP50 and DEHP250, and for 1, 2, 3, and 4 weeks of exposure (Figure 5a). The number of overlapped DEGs for 1, 2, 3, and 4 weeks of the repeated DEHP exposure is shown in Figure 5b. Most of the deregulated genes were unique for every type of exposure. The results also show that the number of DEGs that overlap between the different DEHP-exposed groups is highest after 1 week of exposure (*n* = 3), followed by 2 and 4 weeks of exposure (*n* = 2), while there were no overlapping DEGs between different treatment groups after 3 weeks of exposure. The highest number of overlapping DEGs (*n* = 10) was observed between DEHP250 under the basal condition and DEHP250 under the FOR-stimulated condition after 3 weeks of exposure.

The list of the five top-ranked DEGs for every week of DEHP exposure under the basal conditions is given in Table 2, while the list of the five top-ranked FOR-affected genes that were deregulated by DEHP exposure is shown in Table 3. As shown in bold letters, the most deregulated genes in the DEHP50 and DEHP250 groups (fold change greater than 200) in all weeks of exposure were: sulfotransferase family 1A member 3 (*SULT1A3*) and 4 (*SULT1A4*), polycomb group RING finger protein 4 (*COMMD3-BMI1*)*,* eukaryotic translation initiation factor 3 subunit C-like protein (*EIF3CL*), and piggyBac transposable element derived 3 (*PGBD3*). Except *COMMD3-BMI1,* the same genes were highly deregulated (fold change greater than 200) in DEHP50- and DEHP250-exposed and FOR-stimulated HGrC1 cells (Table 3, bold letters).

### 3.3. Gene Ontology (GO) Analysis

**Figure 6:**
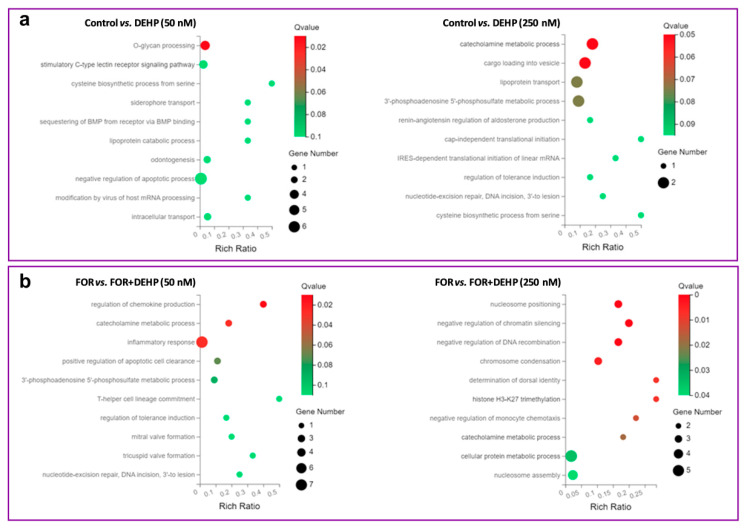
Ten top-ranked biological processes deregulated in DEHP-exposed HGrC1 cells. Summary of deregulated biological processes during four weeks of the repeated exposure to DEHP50 and DEHP250 under the (**a**) basal and (**b**) FOR-stimulated conditions. A greater rich factor represents a greater degree of enrichment. A Q value represents a corrected *p* value. A Q value ≤ 0.05 is regarded as a significant enrichment.

The enriched biological process in the DEHP50 group was O-glycan processing, whereas catecholamine metabolism and cargo loading into vesicles were enriched in the DEHP250 group. The inflammatory response, catecholamine metabolism, and chemokine production were the most enriched biological processes in the DEHP50 and FOR-exposed group, whereas nucleosome positioning, the negative regulation of chromatin silencing, and the negative regulation of DNA recombination were found to be enriched in the DEHP250 and FOR-exposed group (Figure 6). The most enriched molecular function in the DEHP50 group was lubricant activity, whereas in the DEHP250 group, aryl sulfotransferase activity was the most enriched activity. In FOR-stimulated HGrC1 cells, the most enriched molecular functions in the DEHP50 group were complement component C1q binding and aryl sulfotransferase activity, while in the DEHP250 group, the most enriched functions were nucleosomal DNA binding, morphogen activity, and aryl sulfotransferase activity (Supplementary Figure S2).

### 3.4. Validation Study of the RNAseq Data by RT-qPCR

**Figure 7:**
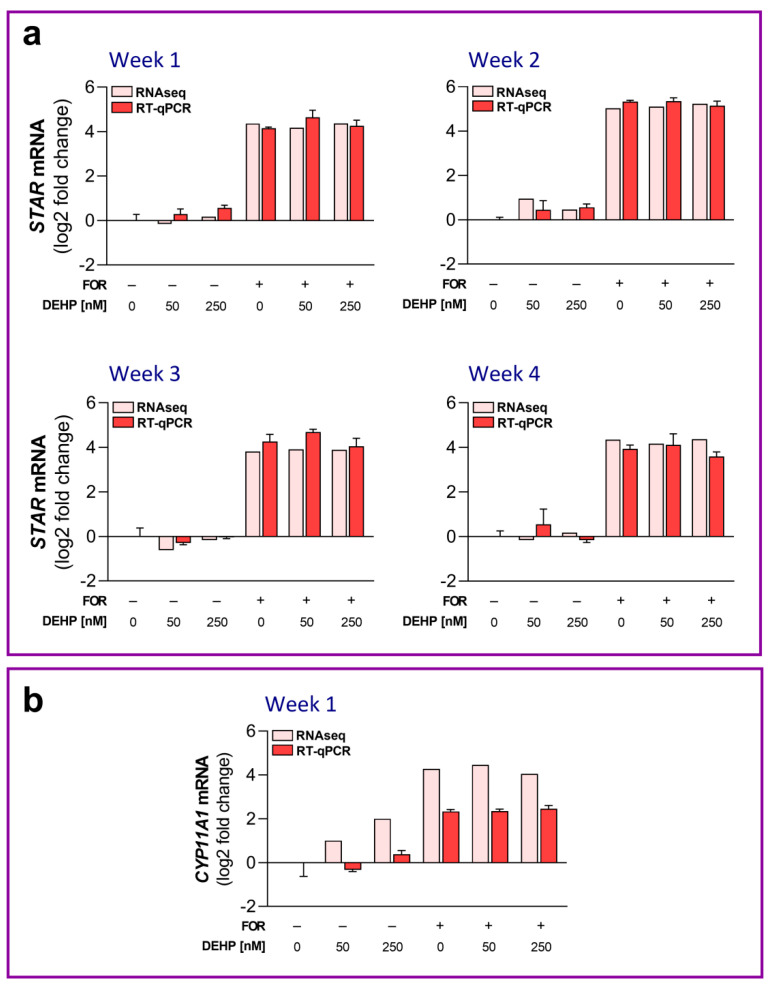
Validation study of the RNAseq data by RT-qPCR. (**a**) The *STAR* mRNA expression levels in HGrC1 cells were analyzed by RT-qPCR after 1, 2, 3, and 4 weeks of the repeated DEHP50 and DEHP250 exposure under the basal and FOR-stimulated conditions and were compared to the RNAseq data. (**b**) The *CYP11A1* mRNA expression levels in HGrC1 cells were analyzed by RT-qPCR after 1 week of the repeated DEHP50 and DEHP250 exposure under the basal and FOR-stimulated conditions and were compared to the RNAseq data. The results are expressed relative to the control that was set as 0 in each week of exposure. In the RT-qPCR experiments, each data point represents the mean ± SEM of three independent experiments.

Next, we performed RT-qPCR to validate the RNAseq data. The samples used for the RNAseq were subjected to RT-qPCR with primer pairs specific for *STAR* and cytochrome P450 side chain cleavage enzyme (*CYP11A1*). We chose these genes since the treatment with FOR induced their expression in HGrC1 cells and because of their important role in steroidogenesis. The expression of *STAR* was validated throughout all four weeks of exposure, whereas *CYP11A1* expression was validated after 1 week of DEHP exposure since the FDR value for FOR stimulation was <0.001 in that week. The expression profiles of the selected genes evaluated by RT-qPCR were consistent with the patterns of expression revealed by the RNAseq (Figure 7a,b). The results were considered to be a technical validation of the DEG analysis.

### 3.5. Effects of the Long-Term Low-Level DEHP Exposure on SULT1A3 and SULT1A4 Expression in HGrC1 Cells under the Basal and FOR-Stimulated Conditions

**Figure 8:**
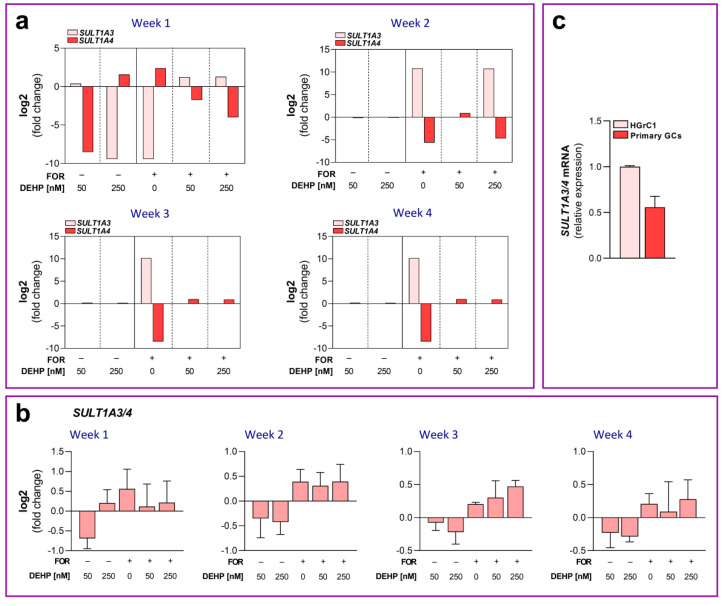
Expression of *SUL1A3* and *SULT1A4* mRNA in DEHP-exposed HGrC1 cells. (**a**) RNAseq data on *SUL1A3* and *SULT1A4* expression in HGrC1 cells after 1, 2, 3, and 4 weeks of the repeated DEHP50 and DEHP250 exposure under the basal and FOR-stimulated conditions. (**b**) RT-qPCR was used to evaluate *SULT1A3/4* mRNA expression in HGrC1 cells. Results are expressed relative to the control that was set as 0 in each week of exposure. (**c**) RT-qPCR was used to evaluate *SULT1A3/4* mRNA expression in the primary culture of human granulosa cells and HGrC1 cells. Results are expressed relative to the expression level in HGrC1 cells that was set as 1 in each week of exposure. In RT-qPCR experiments, each data point represents the mean ± SEM of three independent experiments.

The RNAseq data indicate that the most pronounced effect of the long-term repeated DEHP exposure on HGrC1 cells relates to the changes in the expression of the two genes belonging to the sulfotransferase family, namely, *SULT1A3* and *SULT1A4*. The results show that DEHP50 decreased *SULT1A4* mRNA expression, whereas DEHP250 decreased *SULT1A3* and increased *SULT1A4* mRNA levels only after one week of exposure. FOR decreased *SULT1A3* mRNA levels only after the first week of exposure, whereas it increased mRNA expression of the same gene after 2, 3, and 4 weeks of exposure. In contrast to *SULT1A3,* FOR increased the mRNA expression of *SULT1A4* after one week of exposure but decreased its expression after 2, 3, and 4 weeks. DEHP50 and DEHP250 oppose the effect of FOR on the expression of *SULT1A3* and *SULT1A4* in each week of exposure (Figure 8a). Given the great similarity between the *SULT1A3* and *SULT1A4* genes (>99% identity) [25], we were not able to design specific primers for RT-qPCR. We designed primers that amplify both mRNAs and analyzed the effect of DEHP exposure on *SULT1A3/4* expression. Using these common *SULT1A3/4* primers, we were not able to detect any differences between DEHP50, DEHP250, and FOR compared to the control or between HGrC1 cells stimulated with FOR alone and the cells exposed to the combination of DEHP and FOR (Figure 8b). We also analyzed the expression of *SULT1A3/4* in the primary human cumulus granulosa cells obtained from women undergoing IVF procedure. The results show that *SULT1A3/4* is expressed in primary human granulosa cells, although with a somewhat lower level of expression than in the HGrC1 cells (Figure 8c).

## 4. Discussion

In this study, we applied a model of long-term exposure of human granulosa cells to two environmentally relevant concentrations of DEHP. This model offers a unique approach of prolonged, repeated exposure of human granulosa cells to DEHP and the measurement of dynamic changes in gene responses during each week of the DEHP treatment. This approach advances our understanding of the real-life impact of DEHP on the function of human granulosa cells. Using this model, we demonstrated that the long-term exposure of human granulosa cells to low levels of DEHP does not alter steroidogenesis. However, the RNAseq analysis revealed several genes and functions that were affected by the long-term low-level DEHP exposure. Among them, *SULT1A3* and *SULT1A4* emerged as the most prominent targets of DEHP in unstimulated HGrC1 cells as well as in cells challenged with FOR.

The results on estradiol and progesterone production obtained in this study indicate that DEHP does not affect steroidogenesis in human granulosa cells. Others have also studied the effect of DEHP on steroidogenesis in human granulosa cells. It has been shown that only a high concentration of DEHP decreased estradiol production in the FSH-stimulated KGN cells [12], whereas human exposure-relevant concentrations ranging from 1 nM to 100 nM DEHP lowered progesterone but not estradiol production in the primary culture of human granulosa cells [13]. A similar concentration of 10 nM DEHP did not change the estradiol levels in primary human granulosa cells [5]. It seems that a short exposure to low levels of DEHP can alter progesterone production in the primary culture of human granulosa cells. We do not know if the short-term DEHP exposure changes steroidogenesis in HGrC1. Since the repeated long-term exposure does not alter steroidogenesis, we can assume that DEHP would not change steroidogenesis after a short exposure of HGrC1 cells. It is possible that the primary culture of human granulosa cells is more sensitive to DEHP and that a lower level of exposure to this ED is sufficient to trigger an effect on steroidogenesis in these cells.

Despite the lack of effect on steroidogenesis, this study revealed that DEHP affects several other genes and functions in HGrC1 cells. We observed noticeable dynamic week- and concentration-specific changes in gene expression after repeated DEHP exposure. Some DEGs were only affected by one concentration of DEHP, while others were affected by both concentrations of this ED. Moreover, the majority of DEGs that were affected by DEHP after one week of exposure were not changed in the other weeks. A few DEGs, such as *SULT1A3*, *SULT1A4*, and *PGBD3,* were affected by both concentrations of DEHP in one or more weeks of exposure. A similar dynamic week- and concentration-specific effect of DEHP occurred in FOR-stimulated HGrC1 cells as well. Only a small fraction of genes whose expression was changed by FOR were also affected by DEHP. In the first week, 9 out of 743 FOR-regulated genes were affected by both concentrations of DEHP, including *SULT1A3*, *SULT1A4*, and *PGBD3*. After 2 weeks of exposure, only *PGBD3* emerged as a DEG, whereas *PGBD3*, *SULT1A3*, and *SULT1A4* appeared as DEGs after 3 weeks. Only *SULT1A3* emerged as a commonly regulated DEG after 4 weeks of exposure. In line with these diverse changes in mRNA expression, DEHP also caused week- and concentration-specific changes in the enriched biological processes. Out of all enriched biological processes, catecholamine metabolism can be selected as the biological process enriched in most experimental groups, including the DEHP250 group and the FOR+DEHP50 and FOR+DEHP250 groups.

The results of this study suggest that *SULT1A3* and *SULT1A4* and catecholamine metabolism could be the novel and important targets of DEHP in human granulosa cells. The expression and the role of *SULT1A3* and *SULT1A4* have been investigated in various studies. *SULT1A3* has undergone gene duplication producing two genes: *SULT1A3* and *SULT1A4*. These two genes share 99.9% identical nucleotide sequences and encode the identical enzyme [26]. *SULT1A3* is absent in the human liver but is present in the human small intestine, kidney, lung [27], and brain [28]. This enzyme catalyzes the sulfate conjugation of dopamine and other catecholamines. It was also shown that *SULT1A3/4* are highly expressed in tumor tissue [29]. In hepatocellular carcinoma, *SULT1A3/4* promotes epithelial to mesenchymal transition, migration, and invasion after dopamine activation [30].

This study has revealed several novel findings. The first finding is that *SULT1A3* and *SULT1A4* are expressed in HGrC1 and the primary human granulosa cells, thus adding ovarian granulosa cells as a novel tissue that expresses these two transcripts. The second important finding is that FOR regulates the expression of *SULT1A3* and *SULT1A4* in HGrC1 cells. FOR increases the cyclic adenosine monophosphate (cAMP) levels in cells, suggesting that the expression of *SULT1A3* and *SULT1A4* is regulated by the cAMP signaling pathway in human granulosa cells. These data add new insight into the regulation of *SULT1A3/4* since only a handful of studies to date have described the signaling involved in *SULT1A3/4* regulation. In the SK-N-MC neuroblastoma cell line, the activation of extracellular signal-regulated kinase 1/2 and calcineurin, but not cAMP, is required for the induction of *SULT1A3/4* by dopamine [31]. It was also shown that *SULT1A3* can be induced by glucocorticoid dexamethasone and that this induction depends on the level of the glucocorticoid receptor in the HepG2 hepatocellular carcinoma cell line [32]. It is interesting that FOR shows an opposite effect on the expression of *SULT1A3* and *SULT1A4* mRNA. When *SULT1A3* mRNA is upregulated, the mRNA of *SULT1A4* is downregulated and vice versa in FOR-stimulated HGrC1 cells. This suggests that cAMP may have a bidirectional effect on the expression of these two mRNAs, or there may be some compensatory mechanism that maintains the steady level of *SULT1A3* protein in cells. This specific opposite pattern of expression of *SULT1A3* and *SULT1A4* is preserved throughout all four weeks of exposure. The third important and novel finding in this study is that the expression of *SULT1A3* and *SULT1A4* is sensitive to DEHP exposure. Besides catecholamines, SULT1A3 can catalyze different molecules, such as flavonoids [33] or different drugs [29,34]. Changes in *SULT1A3* and *SULT1A4* expression after DEHP exposure may indicate that these enzymes are involved in the metabolism of this ED in human granulosa cells. However, we have noticed that DEHP, in most cases, opposes the effect of FOR on *SULT1A3* and *SULT1A3* expression in HGrC1 cells. When FOR upregulates *SULT1A3* or *SULT1A4*, DEHP prevents this stimulation and vice versa. This may also indicate the interference of DEHP in FOR-induced signaling pathways that affect the expression of these two transcripts.

Although this study provides important findings regarding the expression and the possible role of *SULT1A3* and *SULT1A4* in human granulosa cells, it also has several shortcomings. We designed specific primers for *SULT1A3* and *SULT1A4*; however, due to very high sequence homology between these two transcripts, we could not obtain the isoform-specific product in RT-qPCR. The product of the *SULT1A4*-specific primers could not be detected in RT-qPCR because these primers anneal poorly to the specific *SULT1A4* sequence. The product of the *SULT1A3*-specific primers showed the same pattern of expression as the product of the primer pair that recognized both mRNA forms. Therefore, the lack of specific primer pairs prevented the confirmation of the data obtained from the RNAseq analysis. The primer pair that recognized both mRNA forms did not recapitulate the RNAseq data. This was an expected finding since *SULT1A3* and *SULT1A4* mRNA show an opposite pattern of expression in human granulosa cells, and the primers will always pick up the mRNA with a higher level of expression. For example, an increase in FOR-induced *SULT1A3* expression in weeks 3 and 4 could not be detected by RT-qPCR due to the presence of *SULT1A4* transcript in the control group. Furthermore, we conducted the experiments in HGrC1 cells, which are different from the primary culture of human granulosa cells. In HGrC1cells, FSH was unable to simulate granulosa cell steroidogenesis, thus adding some uncertainty as to whether FSH can activate its receptor and trigger the FSH-dependent action in HGrC1 cells. To avoid a possible issue with FSH, we had to use FOR, which can mimic some, but not all, of the FSH-mediated actions in human granulosa cells. The primary culture of human granulosa cells routinely responds to FSH; however, these cells show a limited lifespan in culture and cannot be used as the model of the long-term exposure applied in this study.

## 5. Conclusions

In this study, we reveal that low levels of DEHP do not alter steroidogenesis after long-term exposure but interfere with a diverse number of other functions in HGrC1 human granulosa cells. *SULT1A3* and *SULT1A4*, which are involved in catecholamine metabolism, emerged as an important and promising target of the low-level DEHP exposure in human granulosa cells. The work to follow should explore in-depth the role of *SULT1A3* and *SULT1A4* in human granulosa cells and their relationship with DEHP. This knowledge could have a significant impact on our understanding of how DEHP affects human reproductive health.

## Figures and Tables

**Table 1:** Primer sequences used for RT-qPCR analysis.

mRNA (Human)	Forward and Reverse Primer
***SULT1A3/4***	F: 5′-GATCAGAAGGTCAAGGTGGT-3′ R: 5′-TTCCATACGGTGGAAATGGT-3′
***CYP11A1***	F: 5′-GGAGACGGGCACACACAAA-3′ R: 5′-CCCTGTAAATCGGGCCATACT-3′
***STAR***	F: 5′-CGAAGAACCACCCTTGAGAGAA-3′ R: 5′-AGCATTGTTTCCTGGCAAATG-3′
***GAPDH***	F: 5′-CAAGGCTGTGGGCAAGGT-3′ R: 5′-GGAAGGCCATGCCAGTGA-3′

**Table 2:** The top five DEGs after 1, 2, 3, and 4 weeks of the repeated DEHP50 and DEHP250 exposure under the basal conditions.

	Control vs. DEHP (50 nM)			Control vs. DEHP (250 nM)		
Week	Gene Symbol	Fold Change	FDR	Gene Symbol	Fold Change	FDR
**1**	***SULT1A4***	−37	1.63 × 10^−24^	***SULT1A3***	−692	1.72 × 10^−46^
	*LOC101929601*	179	9.03 × 10^−19^	*CORO7-PAM16*	−79	1.06 × 10^−13^
	*USP17L15*	91	3.92 × 10^−7^	*PGBD3*	−68	2.1 × 10^−6^
	*PGBD3*	−68	5.68 × 10^−7^	*TMEM189-UBE2V1*	55	3.41 × 10^−7^
	*HSPE1-MOB4*	47	4.82 × 10^−10^	*LOC107986353*	−33	1.31 × 10^−5^
**2**	*JMJD7-PLA2G4B*	−112	3.76 × 10^−18^	***COMMD3-BMI1***	−550	1.56 × 10^−97^
	*CEMP1*	100	2.65 × 10^−5^	***EIF3CL***	275	1.14 × 10^−27^
	*FSBP*	−49	2.11 × 10^−12^	*CEMP1*	188	5.29 × 10^−11^
	*RGPD2*	−40	7.9 × 10^−14^	*TMEM189-UBE2V1*	80	9.88 × 10^−11^
	*TMEM189-UBE2V1*	40	8.96 × 10^−5^	*PGBD3*	52	1.01 × 10^−10^
**3**	***COMMD3-BMI1***	403	6.37 × 10^−78^	*WRB-SH3BGR*	−105	6.45 × 10^−6^
	*CEMP1*	−115	3.04 × 10^−7^	*ZNF559-ZNF177*	40	5.55 × 10^−5^
	*HSFX2*	−60	1.29 × 10^−5^	*C4A*	−38	1.30 × 10^−9^
	*BCL2L2-PABPN1*	−54	1.30 × 10^−5^	*TBC1D3H*	4.35	4.05 × 10^−5^
	*LOC107986353*	22	2.99 × 10^−4^	*TIAF1*	3.98	5.36 × 10^−9^
**4**	***EIF3CL***	627	1.87 × 10^−66^	***EIF3CL***	553	1 × 10^−59^
	***COMMD3-BMI1***	225	3.7 × 10^−39^	***COMMD3-BMI1***	276	1.59 × 10^−49^
	*PGBD3*	121	7.6 × 10^−13^	***PGBD3***	239	2.58 × 10^−27^
	*CEMP1*	99	1.01 × 10^−5^	*CKMT1A*	−87	1.81 × 10^−7^
	*POC1B-GALNT4*	−76	8.32 × 10^−21^	*TMEM140*	44	3.84 × 10^−4^

**Table 3:** The top five DEGs after 1, 2, 3, and 4 weeks of the repeated DEHP50 and DEHP250 exposure under the FOR-stimulated conditions.

		Control vs. FOR		FOR vs. DEHP (50 nM)		FOR vs. DEHP (250 nM)	
Week	Gene Symbol	Fold Change	FDR	Fold Change	FDR	Fold Change	FDR
**1**	***SULT1A3***	−692	8.16 × 10^−40^	1638	1.06 × 10^−90^	1675	3.96 × 10^−96^
	*CDRT4*	−134	2.7 × 10^−14^	149	1.37 × 10^−14^	58	1.16 × 10^−5^
	*LOC112268437*	−62	4.38 × 10^−11^	129	4.34 × 10^−21^	79	4.86 × 10^−13^
	***PGBD3***	3.95	3.03 × 10^−11^	−269	4.16 × 10^−26^	−269	3.9 × 10^−29^
	***EIF3CL***	7.46	2.75 × 10^−58^	−1194	1.86 × 10^−111^	−6.22	7.8 × 10^−60^
**2**	*ERV3-1-ZNF117*	−126	5.75 × 10^−37^	104	1.25 × 10^−27^	77	2.11 × 10^−20^
	*PGBD3*	88	8.96 × 10^−21^	−176	2.21 × 10^−17^	−176	1.02 × 10^−18^
	*ATP5MF-PTCD1*	40	1.11 × 10^−11^	−40	3.99 × 10^−9^	−40	5.01 × 10^−10^
	*POC1B-GALNT4*	−27	6.38 × 10^−8^	40	3.34 × 10^−9^	31	1.26 × 10^−7^
	*TPTEP2-CSNK1E*	2.86	4.77 × 10^−7^	−3.45	5.84 × 10^−7^	−2.25	3.85 × 10^−4^
**3**	***SULT1A3***	1154	9.41 × 10^−85^	−1154	1.05 × 10^−77^	−1154	8.19 × 10^−78^
	***SULT1A4***	−357.5	8.11 × 10^−51^	699	6.43 × 10^−95^	670.5	3.95 × 10^−92^
	*POC1B-GALNT4*	−20	2.17 × 10^−6^	14	8.81 × 10^−4^	32	3.97 × 10^−9^
	*C4B_2*	18	1.23 × 10^−5^	−18	8.86 × 10^−5^	−18	7.79 × 10^−5^
	*PGBD3*	−6.63	7.3 × 10^−9^	6.42	1.27 × 10^−7^	4.52	1.75 × 10^−4^
**4**	***SULT1A3***	571	7.28 × 10^−4^	−571	1.11 × 10^−39^	−575	7.91 × 10^−40^
	*EIF3CL*	517	4.45 × 10^−58^	2.26	4.17 × 10^−19^	2.357	5.13 × 10^−25^
	*C4A*	−27	3.58 × 10^−8^	19	3.24 × 10^−4^	80	2.84 × 10^−23^
	*PHOSPHO2-KLHL23*	−26	2.36 × 10^−6^	62	4.63 × 10^−13^	27	2.27 × 10^−5^
	*RPL36A-HNRNPH2*	−9.89	2.24 × 10^−76^	6.76	4.81 × 10^−42^	3.89	4.59 × 10^−16^

## Data Availability

The data that support this study will be shared upon reasonable request to the corresponding author.

## Supplementary Materials

The following supporting information can be downloaded at: https://www.mdpi.com/article/10.3390/cells11152304/s1, Figure S1: CYP19A1 and STAR protein expression HGrC1 cells after the long-term repeated exposure to DEHP. Western blot analysis was used to analyze (a) CYP19A1 and (b) STAR protein expression after 1, 2, 3, and 4 weeks of the repeated exposure to DEHP50 and DEHP250 under the (a) basal and (b) FOR-stimulated conditions. Representative Western blot images are shown. Results are expressed relative to the vehicle-treated control that was set as 1 in each week. Each data point represents the mean ± SEM of 2–3 independent experiments. Different superscript letters indicate statistically significant differences among treatment groups (*p* < 0.05); Figure S2: Ten top-ranked molecular functions deregulated in HGrC1 cells after the long-term repeated exposure to DEHP. Summary of deregulated molecular functions during all 4 weeks of exposure to DEHP50 and DEHP250 under the (a) basal and (b) FOR-stimulated conditions. A greater rich factor represents a greater degree of enrichment. A Q value represents corrected *p* value. Q value ≤ 0.05 is regarded as a significant enrichment.
