# Supplementary material for: Impact of In Vitro Long-Term Low-Level DEHP Exposure on Gene Expression Profile in Human Granulosa Cells
Source: Cells. 2022 Jul 27;11(15):2304. doi: 10.3390/cells11152304 (PMC9332775; doi:10.3390/cells11152304)
Supplement: Supplementary file 1 [file cells-11-02304-s001.zip › cells-1799414-supplementary.pdf]

SUPPLEMENTARY MATERIAL

Impact of *In Vitro* Long-Term Low-Level DEHP Exposure on Gene Expression Profile in Human Granulosa Cells

Dragana Samardzija Nenadov[1], Kristina Pogrmic-Majkic[1]*, Biljana Tesic[1], Dunja Kokai[1], Svetlana Fa Nedeljkovic[1], Bojana Stanic[1] and Nebojsa Andric[1]

[1]University of Novi Sad, Faculty of Sciences, Department of Biology and Ecology, Serbia

*Corresponding author:
Kristina Pogrmic-Majkic, Ph.D.
University of Novi Sad, Faculty of Sciences, Department of Biology and Ecology
Trg Dositeja Obradovica 2, 21000 Novi Sad, Serbia
phone: +381 21 485 2675, fax: +381 21 450 620
email: kristina.pogrmic@dbe.uns.ac.rs

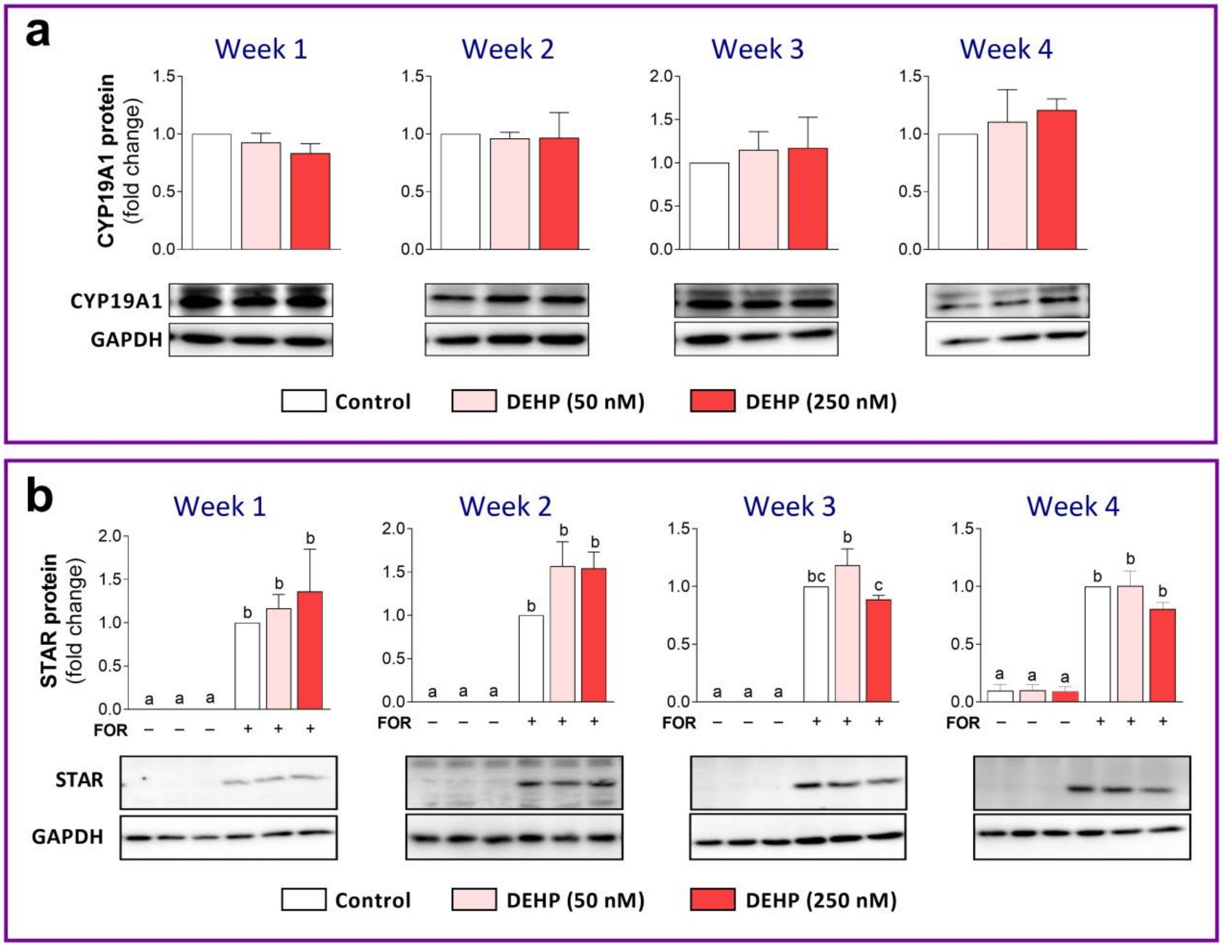

Figure S1. CYP19A1 and STAR protein expression HGrC1 cells after the long-term repeated exposure to DEHP. Western blot analysis was used to analyze (a) CYP19A1 and (b) STAR protein expression after 1, 2, 3, and 4 weeks of the repeated exposure to DEHP50 and DEHP250 under the (a) basal and (b) FOR-stimulated conditions. Representative Western blot images are shown. Results are expressed relative to the vehicle-treated control that was set as 1 in each week. Each data point represents the mean ± SEM of 2-3 independent experiments. Different superscript letters indicate statistically significant differences among treatment groups ($p < 0.05$).

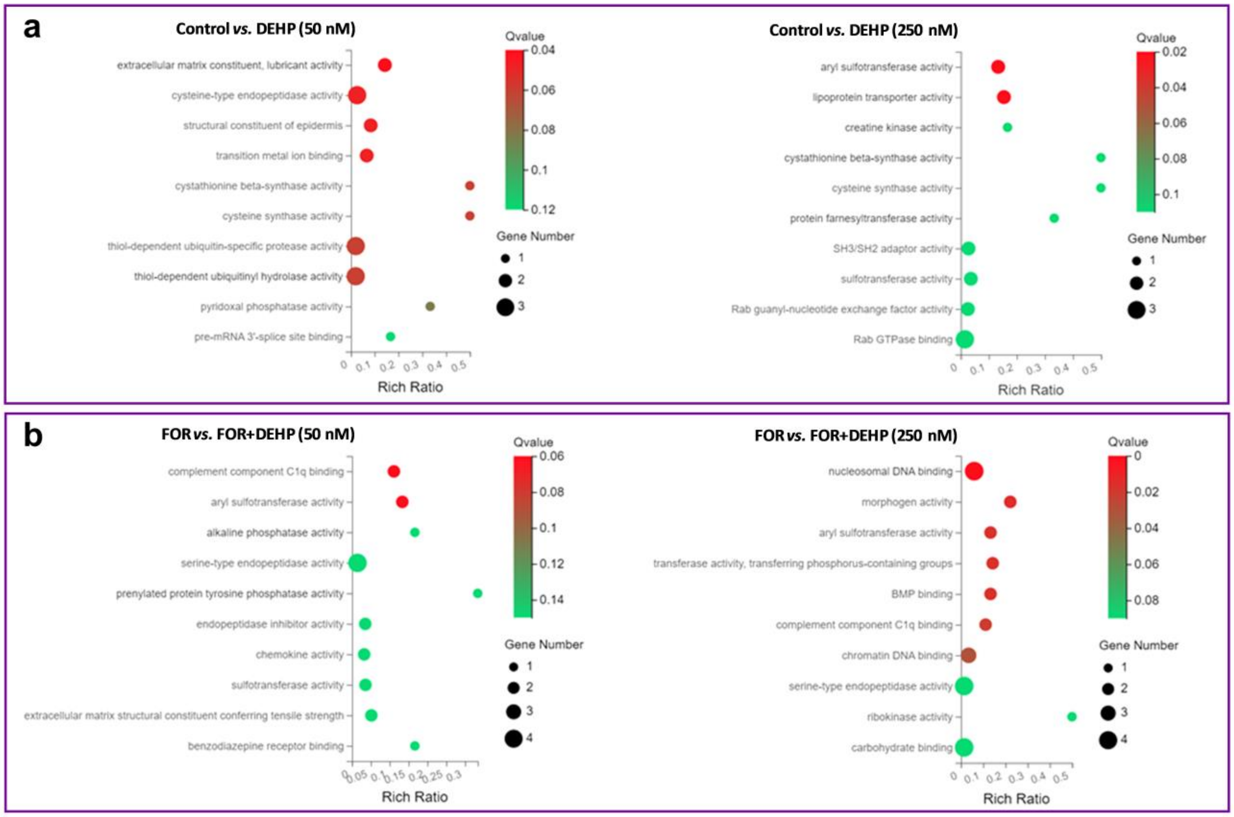

Figure S2. Ten top-ranked molecular functions deregulated in HGrC1 cells after the long-term repeated exposure to DEHP. Summary of deregulated molecular functions during all 4 weeks of exposure to DEHP50 and DEHP250 under the a) basal and b) FOR-stimulated conditions. A greater rich factor represents a greater degree of enrichment. A Q value represents corrected *p* value. Q value ≤ 0.05 is regarded as a significant enrichment.
